# Verification of the formulation and efficacy of *Danggui Buxue Tang *(a decoction of *Radix Astragali *and *Radix Angelicae Sinensis*): an exemplifying systematic approach to revealing the complexity of Chinese herbal medicine formulae

**DOI:** 10.1186/1749-8546-2-12

**Published:** 2007-11-29

**Authors:** Qiutao Gao, Jun Li, Jerry Ka Hei Cheung, Jinao Duan, Anwei Ding, Anna Wing Han Cheung, Kuijun Zhao, Winnie Zhuoming Li, Tina Tingxia Dong, Karl Wah Keung Tsim

**Affiliations:** 1 Department of Biology and the Center for Chinese Medicine, Hong Kong University of Science and Technology, Clear Water Bay Road, Hong Kong SAR, China; 2 Nanjing University of Traditional Chinese Medicine, 138 Xianlin Dadao, Xianlin University Town, Nanjing 210046, China; 3 Beijing Friendship Hospital (an affiliate of the Capital Medical University), 95 Yongan Road, Beijing 100050, China

## Abstract

This article exemplifies a systematic approach to revealing the complexity of Chinese herbal medicine formulae through three levels of scientific research: standardization of herbs, verification of ancient formulae and mechanism studies. We use *Danggui Buxue Tang *(*DBT*) as an example for this approach. Among thousands of traditional Chinese medicine herbal formulae, almost all of which consist of multiple herbs, *DBT *is one of the simplest. Containing only two herbs, namely *Radix Astragali *(*RA*) and *Radix Angelicae Sinensis *(*RAS*), *DBT *is traditionally used to treat ailments in women. The weight ratio of *RA *to *RAS *in *DBT *was prescribed to be 5:1 as early as in 1247 AD. In addition to advanced chemical analysis of herbal constituents, DNA genotyping techniques have been developed for reliable standardization of *RA *and *RAS*. Chemical evaluation shows that main active constituents in *DBT*, including astragaloside IV, calycosin, formononetin and ferulic acid, were most abundant after extraction at the *RA *to *RAS *ratio of 5:1, whereas other tested *RA *to *RAS *ratios only gave sub-optimal levels of the active constituents. Biological evaluation indicates that bioactivities of *DBT*, e.g. immuno-modulatory, oesteotropic and estrogenic effects are also best exerted at the *RA *to *RAS *ratio of 5:1. Correlation analysis demonstrates statistically significant relationship between the tested chemical constituents and tested bioactivities. Up- and down-regulation of expression of some genes as potential biomarkers has been detected by using gene chip technology. This systematic approach on the basis of herbal standardization, chemical and biological verification and mechanism studies, as exemplified in this article, will be useful to reveal the complexity of not only *DBT *but also other Chinese medicine herbal formulae.

## Background

Traditional Chinese medicine (TCM) has been used to improve the well-being of the Chinese people for thousands of years. TCM products, many of which were raw materials, made up only 3% of the 16 billion USD international herbal medicine market in 2004 [1,2]. Since the market opening-up of China, international pharmaceutical companies have been gaining a market share in both conventional and herbal medicine products in China. In the 21^st ^century, TCM products should meet stringent international quality and safety standards through modernization; otherwise they will lose their competitiveness.

Standardization as the basis of modernization and internationalization of TCM is the key to ensure the safety and efficacy of TCM products. At present, lack of standardization in TCM products impedes the development of TCM. For instance, it is common that different herbs have the same name or a single herb has different names in the market. Some herbs cultivated in different regions or harvested in different seasons may vary considerably in their chemical and biological properties. Most of the TCM products do not have specific biomarkers. TCM is traditionally administered in the form of a decoction with a combination of different herbs. The complexity of biological effects of the interactions among different compounds within a decoction complicates experimental studies to reveal the action mechanisms.

Among thousands of TCM formulae, *Danggui Buxue Tang *(*DBT*) is one of the simplest. The formula consists of only two herbs: *Radix Astragali *(*RA, Huangqi*) and *Radix Angelicae Sinensis *(*RAS, Danggui*) in a weight ratio of 5:1. According to a traditional method, the herbs are boiled together in two bowls of water at moderate heat until the final volume has been reduced to one bowl [2]. In a book entitled *Neiwaishang Bianhuo Lun *in 1247 AD, *DBT *was first described by Li Dongyuan, one of the four well-known TCM physicians during the Jin and Yuan Dynasties in China.

In this review, we summarize recent findings of *DBT *to exemplify a systematic approach to revealing the complexity of Chinese herbal medicine formulae through three levels of scientific research: standardization of raw materials, verification of ancient formulae and mechanism studies.

### Standardization of *Radix Astragali *and *Radix Angelicae *Sinensis

**Figure 1:**
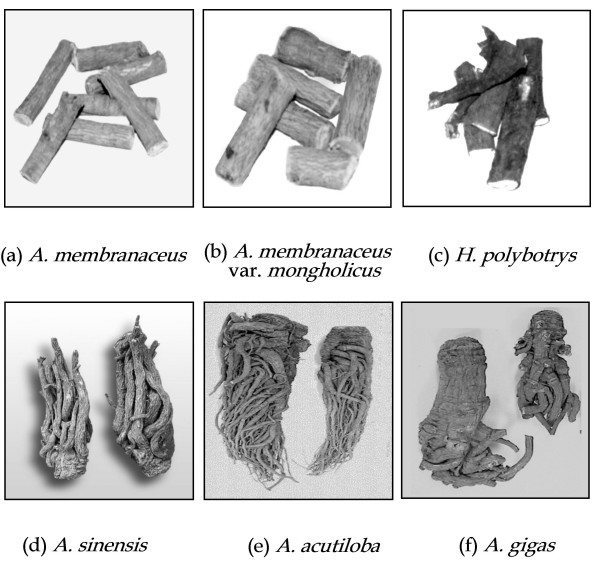
**The authentic sources of *RA *and *RAS***. **(a) ***A. membranaceus *and **(b) ***A. membranaceus *var. *mongholicus *are the sources for *RA*. **(c) ***H polybotrys *is a common substitute for *RA*. **(d) ***A. sinensis *is the source for *RAS*. **(e) ***A. acutiloba *and **(f) ***A. gigas *are also sold as raw materials for *RAS *in the markets.

A reliably reproducible chemical composition of *DBT *is a prerequisite in delineating the biological effects of this Chinese medicine preparation. The quality of *RA *and *RAS *may be considerably influenced by weather, geographic location, soil conditions, and the methods of cultivation and processing. Some Chinese medicinal materials with excellent quality are only produced in certain regions of China which are often referred to as 'the best growth region' or '*Didao*'. Therefore, how to authenticate and choose the best *RA *and *RAS *plays a critical role in ensuring the quality of *DBT *(Figure 1).

#### Radix Astragali

*Astragalus *L. (Leguminosae) is a large genus with over 2,000 species worldwide and more than 250 sections in angiosperm family *Fabaceae *(subfamily *Papilionoideae*). Both listed as the botanical sources of *RA *in Chinese Pharmacopoeia (2005) [3], *Astragalus membranaceus *(Fisch.) Bunge and *Astragalus membranaceus *(Fisch.) Bunge var. *mongholicus *(Bunge) P.K. Hsiao [4,5] are the most commonly used *RA*. The morphological appearances and chemical properties of *RA *and its adulterants show a remarkable resemblance [6-8]. The DNA sequences of 5S rRNA spacer, ITS and 18S rRNA coding region were determined and compared among ten *Astragalus *taxa [6-8]. With neighbor-joining and maximum parsimony analyses, phylogenetic trees were mapped according to their sequence diversity. *A. membranaceus *and *A. membranaceus *var.*mongholicus *have the highest sequence homology. The common substitute of *RA *in some parts of China is the roots of *Hedysarum polybotrys *which has very different genetic makeup from that of the *Astragalus *species [9] (Figure 1).

**Figure 2:**
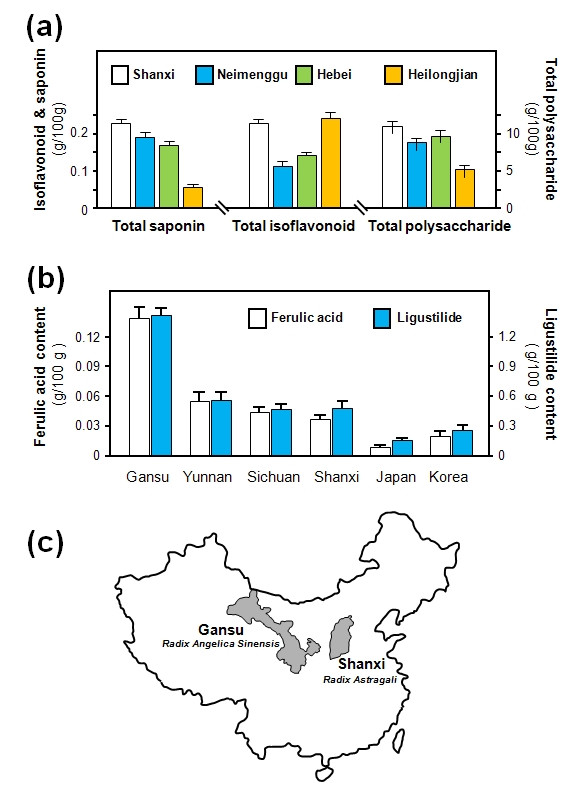
**Determination of the active constituents in *RA *and *RAS***. **(a) **Amounts of total saponin, total isoflavonoid and total polysaccharides were determined in *RA *collected from various regions in China. **(b) **Amounts of ferulic acid and ligustilide were determined in *RAS *collected from various regions and countries. The roots of *A. sinensis *collected from Gansu, Yunnan, Sichuan and Shanxi, China were used. The roots of *A. acutiloba *from Hokkaido, Japan and *A. gigas *from Sokcho, Korea were used. Values are in g/100 g of dry herbal materials with means ± SEM, n = 10. **(c) ***RAS *from Gansu, China and *RA *from Shanxi, China should be used for *DBT *preparation.

HPLC and spectrophotometry were used to determine the levels of isoflavonoids, astragalosides, polysaccharides, amino acids and trace elements, which are the main active constituents in different *Astragalus *species and *RA *collected in different seasons and of various ages. The results indicated that *RA *of three years of age from Shanxi, China (Figure 2a) contained the highest amounts of isoflavonoids, saponins and polysaccharides [6,10].

#### Radix Angelicae Sinensis

According to the Chinese Pharmacopoeia (2005) [3], *RAS *is the root of *Angelica sinensis *(Oliv.) Diels (family *Umbellaceae*); however, *Angelica acutiloba *(Sieb. et Zucc.) Kitag. and *Angelica gigas *Nakai, mainly found in Japan and Korea respectively, are also sold as *RAS *in the markets of South East Asia [11-14] (Figure 1). Studies have shown that the three commonly used *Angelica *roots vary in their chemical composition, pharmacological properties and efficacy [9,11]. The 5S-rRNA spacer domains of the three species of *Angelica *were amplified and their nucleotide sequences were determined. The sequence of *A*. *sinensis *is 72.87% and 73.58% identical to those of *A. acutiloba *and *A. gigas *respectively, while *A. acutiloba *and *A. gigas *are 93.57% identical in their sequences [9]. The phylogenetic tree clearly reveals that the three *Angelica *species are divided into two clusters: *A. sinensis *is in one cluster and *A. acutiloba *and *A. gigas *are in another.

The main chemical constituents of *Angelica *roots are ferulic acid, Z-ligustilide, angelicide, brefeldin A, butylidenephthalide, butyphthalide, succinic acid, nicotinic acid, uracil and adenine [9,15-17]. The levels of ferulic acid and Z-ligustilide are often used as chemical markers for the quality control of *Angelica *roots [16]. In *A. sinensis *roots from Gansu, China, the levels of ferulic acid and Z-ligustilide are about ten-fold higher than those of the roots of *A. acutiloba *(from Japan) and *A. gigas *(from Korea) [9,17] (Figure 2b). Su Jing (659 AD) in *Tang Bencao *and Li Shizhen (1596 AD) in *Bencao Gangmu *recorded that *Angelica *roots of two years of age produced in Gansu were the authentic source. *RAS *from Gansu contains about two-fold higher amounts of Z-ligustilide and ferulic acid than those *RAS *from Yunnan, Shanxi or Sichuan, China [9] (Figure 2b). To ensure the best quality of *DBT *decoction, we suggest that standardized *RA *from Shanxi and standardized *RAS *from Gansu should be used in all *DBT *preparations (Figure 2c).

### Verification of the DBT formula

#### Chemical evaluation

Li Dongyuan (1247 AD) documented that *RA *and *RAS *combined at a ratio of 5:1 demonstrated the best efficacy. In a previous study [18], *DBT *was prepared by boiling the herbal mixture under various conditions and the results indicated that the 5:1 ratio indeed provided the maximum levels of active constituents of *DBT*. Furthermore, the levels of active constituents and biological activities of *DBT *extracts were investigated with preparations of *RA *and *RAS *at ratios of 1:1, 2:1, 3:1, 4:1, 5:1, 7:1 and 10:1.

**Figure 3:**
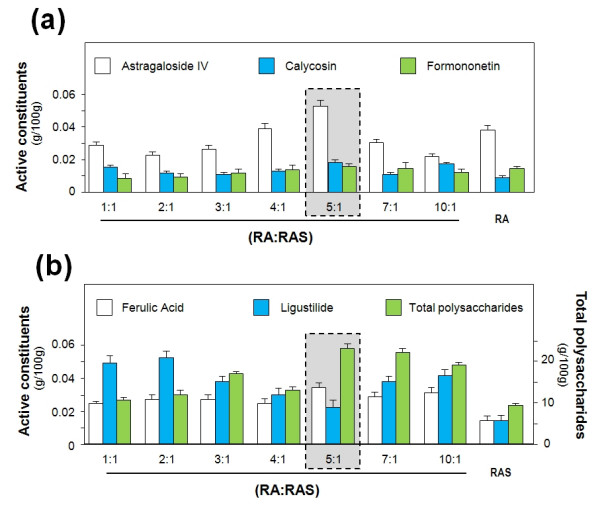
**Chemical constituents in *RA*, *RAS *and *DBT***. **(a) **The amounts of astragaloside IV, calycosin, formononetin in *DBT *of various ratios of *RA *to *RAS*. **(b) **The amounts of Ligustilide, ferulic acid and total polysaccharides in *DBT *of various ratios of *RA *to *RAS*. Values are in mg/g of dry material (normalized by each herb weight) with means ± SEM, n = 5, each with triplicate samples.

Used as chemical markers, the main active constituents in *DBT *include *RA*-derived astragaloside IV, calycosin and formononetin, *RAS*-derived ferulic acid and ligustilide, and total saponins, total flavonoids and total polysaccharides [19]. The detected levels of the chemical markers varied significantly among the seven preparations (Figure 3). The level of astragaloside IV of the 5:1 ratio preparation was the highest, 2-fold higher than the 10:1 ratio preparation which recorded the lowest level [19]. The 5:1 ratio preparation also contained the highest level of calycosin, formononetin, and ferulic acid. As regards the levels of total saponins, total flavonoids and total polysaccharides, the 5:1 ratio *DBT *preparation recorded the highest levels (Figure 3) [19].

There are several possibilities for higher levels of active chemical constituents in *DBT *preparations. Firstly, compounds such as saponins (over 2% in total dry weight) [10,17] in *RA *may help increase the solubility of other compounds extracted from *RAS*. For example, astragaloside increases the solubility of *RAS*-derived ferulic acid and ligustilide. Secondly, ferulic acid and ligustilide are readily oxidized under heat, which means they can be degraded when boiled [9]. However, when *RAS *is boiled together with *RA*, compounds derived from *RA *may prevent this oxidization process, thereby producing a higher yield of ferulic acid and ligustilide in *DBT *preparations. Thirdly, the stability of those active constituents may be improved by having a cocktail of different chemicals. Further research is required for better understanding of this complexity.

#### Biological evaluation

According to TCM theories, *DBT *replenishes *qi *and nourishes *xue *(the blood). *DBT *is therefore used for treating menopausal symptoms [2]. Due to a deficiency of ovarian hormones, especially estrogen, women in menopause often suffer from hot flashes, sweating, anxiety, mood swings and an increased risk for other health problems, such as reduction of bone mineral density and cardiovascular diseases [20]. Apart from a lack of estrogen, the immune system is also involved in the menopausal symptoms. Steroid hormones may modulate the immune response [21] and immune reactions may also regulate the ovarian function [22]. Various bioactivities related to menopausal symptoms, such as osteotropic effect, estrogenic effect, anti-platelet aggregation effect and immuno-modulatory effect have been used to evaluate the functional roles of *DBT*.

**Figure 4:**
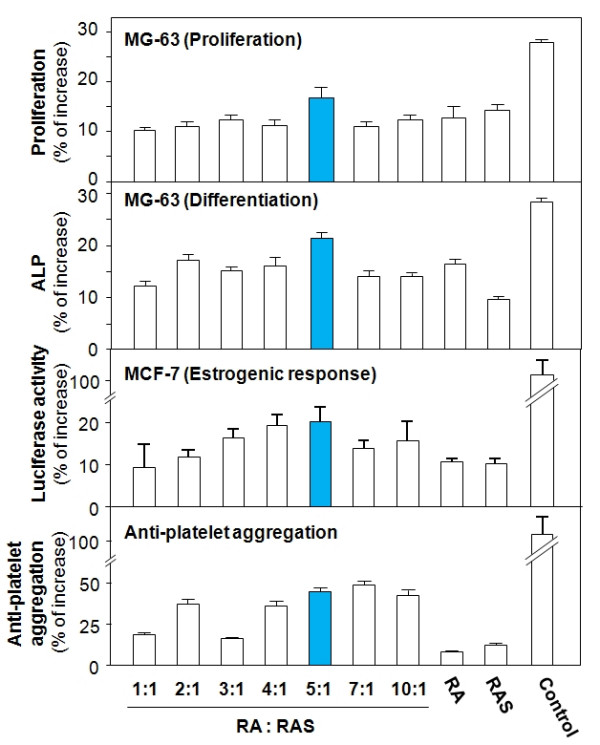
**Biological activities of *RA*, *RAS *and *DBT *of various *RA *to *RAS *ratios**. *RA*, *RAS *and *DBT *of various *RA *to *RAS *ratios were tested for MG-63 cell proliferation (MTT assay), MG-63 cell differentiation (ALP assay), estrogenic response (estrogen promoter) and anti-platelet aggregation activity. The values are means ± SD, n = 5, each with triplicate samples.

*DBT *extract was applied to a cultured human MG-63 osteosarcoma cell. Bone cell proliferation and differentiation were measured by 3-(4, 5-dimethylthioazol-2-yl)-2, 5-diphenyltetrazolium bromide (MTT) assay and alkaline phosphatase (ALP) assay. *DBT *induced both the proliferation and differentiation of osteoblast MG-63 cells in a dose-dependent manner. In both assays, *DBT *showed stronger effects than *RA *or *RAS *alone, In the MTT assay, the 5:1 ratio *DBT *extract stimulated MG-63 cell proliferation, which was 10–20% higher than the extracts of other ratios (Figure 4). For bone cell differentiation, the 5:1 ratio *DBT *preparation induced ALP activity to the highest level among all ratios and showed the strongest osteotropic effect [19].

The estrogenic effects of *DBT *were tested by a cellular reporter system of transcriptional activation of estrogen receptor/promoter. A promoter/reporter construct (pERE-Luc) corresponding to the responsive elements of estrogen receptor was stably transfected into MCF-7 cells. The *DBT *extracts of various ratios were applied onto the cultures for 2 days. Two parameters, namely cell number and promoter activity (luciferase activity), were determined. While *DBT *was not able to alter the proliferation of MCF-7 cells, the estrogen-driven promoter activity was markedly induced by *DBT *(Figure 4); the 5:1 ratio *DBT *showed the strongest effect in inducing the promoter activity than RA, *RAS *alone or the extracts of other ratios [19].

In anti-platelet aggregation assay, the activity of *DBT *in preventing ADP-induced platelet aggregation was determined. The ratios 5:1 and 7:1 *DBT *extracts demonstrated higher levels of activity in preventing platelet aggregation than either *RA*, *RAS *alone or the extracts of other ratios (Figure 4) [19].

In a study of immuno-modulatory effects, *DBT *preparations of various ratios were applied to cultured T-lymphocytes and macrophages. In cultured T-lymphocytes, *DBT *induced markedly cell proliferation, interleukin-2 secretion and the phosphorylation of extracellular signal-regulated kinase (ERK1/2). In addition, the phagocytosis of cultured macrophages was elevated by *DBT *treatment. The immuno-modulatory effects of 5:1 ratio *DBT *were the strongest [23] (Table 1).

**Table 1:** Biological evaluation of *DBT *(*in vitro *studies)

Findings	Model	Treatment	Reference
The 5:1 ratio *DBT *showed stronger effects in stimulating MG-63 cell proliferation and induced ALP activity to the highest level among all groups.	Cultured human MG-63 osteosarcoma cells	*DBT *of various ratios of *RA *and *RAS*, compared with β-estradiol and negative control	Dong *et al*. [19]
The 5:1 ratio *DBT *showed the strongest effect in inducing the estrogen-driven promoter activity than RA, *RAS *alone or the extracts of other ratios.	Cultured MCF-7 cells	*DBT *of various ratios of *RA *and *RAS*, compared with β-estradiol and negative control	Dong *et al*. [19]
The ratios 5:1 and 7:1 *DBT *showed higher levels of activity in preventing platelet aggregation.	ADP induced-platelet aggregation in blood from adult New Zealand white rabbits	*DBT *of various ratios of *RA *and *RAS*, compared with ticlopidine and negative control	Dong *et al*. [19]
*DBT *induced cell proliferation, interleukin-2 secretion and the phosphorylation of extracellular signal-regulated kinase (ERK1/2) in cultured T-lymphocytes. The 5:1 ratio *DBT *showed the strongest immuno-modulatory effects.	Cultured T-lymphocytes and macrophages	*DBT *of various ratios of *RA *and *RAS*, compared with PHA, PMA, Zymosan A and negative control	Gao *et al*. [23]

In addition to the *in vitro *assays, the 5:1 ratio of *RA *and *RAS *in *DBT *was further tested and verified by animal studies. In *DBT*-administrated mice, the 5:1 ratio preparation was the most effective decoction in triggering immune responses [24,25].

The pharmacological studies in animals also suggest that *DBT *has the ability to promote hematopoiesis, to stimulate blood circulation, to prevent osteoporosis and to counter oxidative stress [19,26,27]. Moreover, *DBT *is known to enhance myocardial mitochondria and glutathione status in red blood cells, thereby increasing their resistance to injury induced by oxidative stress [28]. In rats, *DBT *protected against myocardial ischemia-reperfusion injury in a dose-dependent manner [28]. A more potent cardio-protection was demonstrated in *DBT*-treated rats than in rats treated with either extracts of *RA*, *RAS *alone, or a mixture of *RA *and *RAS *(not boiled together). When the mice were administered orally with *DBT*, the serum collected from abdominal aorta was added to an *in vitro *cultivating system of mouse hematopoietic progenitor cells. The decoction-contained serum showed promoting actions to CFU-GM and CFU-E. Once again, the action of the 5:1 ratio *DBT *was 97.81% stronger than that of the 1:1 ratio extract [29,30] (Table 2).

**Table 2:** Biological evaluation of *DBT *(*in vivo *studies)

Findings	Model	Treatment	Reference
*DBT *had significantly higher RBC and Hb levels in both normal and anemic mice than those in *RA*, *RAS *and control.	Kunming mice, male, RBC, Hb	Normal mice in 4 groups: *RA*, *RAS*, *DBT *and control; Anemic mice in 4 groups: *RA*, *RAS*, *DBT *and control	Wu BC *et al*. [24]
*DBT *was the most effective decoction in triggering immune responses.	Kunming mice, RBC, Hb, WBC, Plt, reticulocyte, nucleated cells of bone cavity, weight of pancreas and thymus	Mice in 5 groups: *RA*, *RAS*, *DBT*, *RA*+*RAS *(1:1) and control	Li YK *et al*. [25]
*DBT *alleviated cardiac injury in ischemia reperfusion.	Wister rats (male), amplitudes of LVSP and ± dp/dtmax, arterial pressure, Na+-K+-ATP activity, level of MDA production, cAMP content	Rats in myocardial ischemia reperfusion injury; i.v.	Wu DZ *et al*. [26]
*DBT *increased the levels of RBC, WBC, and BMNC. Some *DBT *promoted the proliferation of BMNC and increased the level of CFU-Mix.	Kunming mice, ICR mice, Balb/c mice, RBC, WBC, reticulocytes and BMNC	Mice in 4 groups: normal, model, *DBT *without polysaccharides, *DBT *with polysaccharides	Ning L *et al*. [27]
*DBT *enhanced myocardial mitochondria and red blood cell glutathione status.	Rats, myocardial mitochondrial status, RBC glutathione status	Rats in 5 groups: *RA*, *RAS*, *DBT*, *RA *+ *RAS *(not boiled together) and control; orally administered	Mak DH *et al*. [28]
*DBT *inhibited growth of GM-CFU, while the decoction-containing serum promoted growth of GM-CFU.	Kunming mice, GM-CFU	*DBT *was administered orally; serum collected from abdominal aorta was added to an *in vitro *cultivating system of mouse hematopoietic progenitor cells.	Zhang YH *et al*. [29]
The decoction-containing serum showed promoting actions to CFU-E. *RA*+*RAS *(5:1) was 97.81% stronger than *RA*+*RAS *(1:1).	Kunming mice, CFU-E	*DBT *was administered orally; serum collected from abdominal aorta was added to an *in vitro *cultivating system of mouse hematopoietic progenitor cells.	Zhang YH *et al*. [30]

### Mechanism studies

#### Correlation between chemical fingerprints and bioactivities of DBT

**Figure 5:**
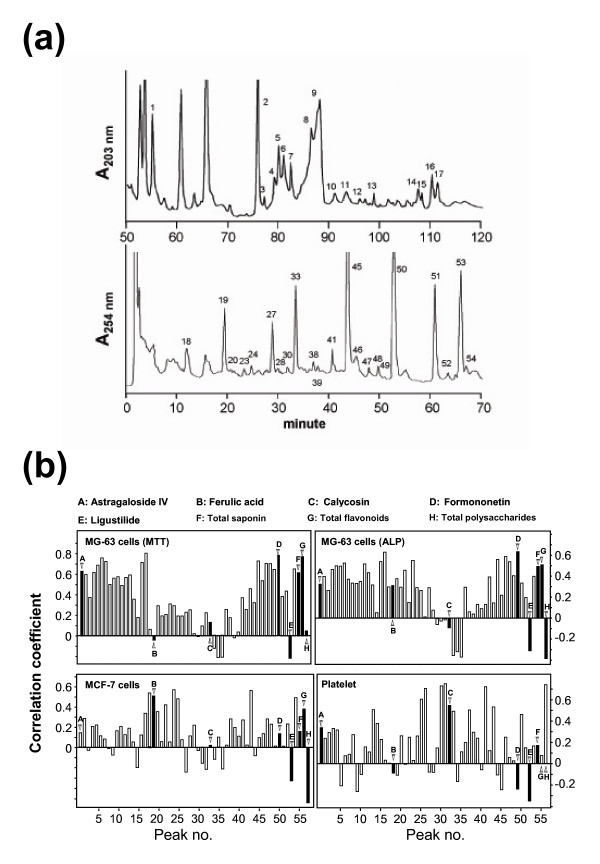
**Correlation coefficients between the data of 57 chemicals and the four bioassays**. **(a) **Fifty-four peaks in typical HPLC fingerprints of *DBT*. In the HPLC fingerprint of 203 nm, astragaloside IV and other 16 peaks had a retention time between 70 to 120 min. In the HPLC fingerprint of 254 nm, ferulic acid, calycosin, formononetin, ligustilide and other 32 peaks had a retention time between 0 to 70 min. The 54 peaks are numbered, where astragaloside IV (1), ferulic acid (19), calycosin (33), formononetin (50) and ligustilide (53) are identified and served as standards. **(b) **The correlation coefficients between the data of 57 chemicals with the four bioassays. The correlation coefficient is in Y-axis and the peak number is in X-axis. Individual chemical markers are indicated by arrowheads and denoted by astragaloside IV (A), ferulic acid (B), calycosin (C), formononetin (D), ligustilide (E), total saponins (F), total flavonoids (G) and total polysaccharides (H). All correlations were tested to be statistically significant (P < 0.05).

Fifty-four chemical peaks were detected in *DBT *extracts by an HPLC analysis (Figure 5a) and a total of over 100 *DBT *extracts from various preparations were analyzed [27]. Among these 54 peaks, the markers for *RA*-derived astragaloside IV, calycosin and formononetin, and for *RAS*-derived ferulic acid and ligustilide were identified. In analysis of correlation, the identified 54 peak areas together with the contents of total saponins, total flavonoids and total polysaccharides were considered as independent variables. The results of the four bioactivities, namely proliferation and differentiation of MG-63 cells, estrogenic property in MCF-7 cells and anti-platelet aggregation activity, were considered as dependent variables. By analyzing the correlation of these two kinds of variables, coefficients of correlation between the HPLC data of the 57 chemicals and the bioassay data of the *DBT *extracts were obtained. The values of the coefficients indicate possible relationship of these chemical peaks with bioactivities, where positive values suggest positive effects of chemicals on bioactivities and negative values suggest negative effects.

In the assay of MG-63 cell proliferation, astragaloside IV, formononetin, total saponins and total flavonoids are correlated with the bioactivities (Figure 5b). In the assay of MG-63 cell differentiation, formononetin, total saponins and total flavonoids are correlated with the bioactivities. In the analysis of estrogen promoter in MCF-7 cells, ferulic acid are correlated with the bioactivities. Calycosin and total polysaccharides were two very important factors in the assay of anti-platelet aggregation. On the other hand, the amount of ligustilide showed negative effects in all bioassays (Figure 5b). Other components of *DBT*, such as those corresponding to peaks 5 to 15, have high correlation coefficients with the bioactivities, but are yet to be identified.

#### Specific estrogenic and immuno-modulatory effects of DBT

The estrogenic effects of *DBT *were investigated by determining the levels of phosphorylation of estrogen receptor α (ERα) and extracellular signal-regulated kinase 1/2 (ERK1/2) in cultured MCF-7 cells. In contrast to estrogen, *DBT *triggered the phosphorylation of ERα and ERK1/2 at both S118 and S167 in a time-dependent manner. Although the activity of the estrogen-responsive element in pERE-Luc stably expressing MCF-7 cells was activated by extracts of either *RA *or *RAS *alone, or by a mixture of *RA *and *RAS*, the phosphorylation of ERα at S167 and of ERK1/2 were only found in *DBT*-treated cultures. Interestingly, the specific estrogenic effects of *DBT *were not only shown in the MCF-7 cells [31].

In cultured T-lymphocytes, the phosphorylation of the ERK 1 (about 42 kDa) and ERK 2 (about 44 kDa) was increased by *DBT *[30]. The induction was transient. An approximately eight-fold increase of ERK phosphorylation was detected 20 minutes after *DBT *was applied, whereas the phosphorylation was undetectable in the cultures treated with extracts of either *RA *or *RAS *alone [31]. Moreover, the phosphorylation of ERK in T-lymphocytes could not be activated by a simple mixture of extracts of *RA *and *RAS*. This result suggests that boiling *RA *and *RAS *together is essential for *DBT *to exert estrogenic effects.

#### Genomics

For decades, scientists mainly isolated pure chemicals from herbal extracts and then screened for biological activities and possible targets. This strategy does not garantee to isolate and/or identify active chemicals from well-known medicinal plants because a single chemical compound may not fully account for the overall effects of herbal extracts. Recent advances in genomics and proteomics have enriched our tool sets to reveal the complex nature of TCM decoctions. An experiment on *DBT*-regulated genes was carried out in our laboratory using gene chip (i.e. microarray) technology. Cultured MG-63 cells were treated with 1 mg/ml of *RA*, *RAS *or *DBT *for 24 hours. The isolated mRNAs were analyzed using microarray, which is a quantitative method to investigate the change of mRNA expression profiles between the control and treatment groups. A total of 8064 genes were screened. Significant changes in gene expression were found after the treatment of *DBT*, *RA *or *RAS *(Table 3). A total of 883 genes were either up or down regulated by *DBT *treatment of which 403 genes were *DBT*-specific; 660 genes were regulated by *RA *treatment of which 172 genes were *RA*-specific; 1,062 genes were regulated by *RAS *treatment of which 473 genes were *RAS*-specific. In addition, 279 genes were commonly regulated by the extracts of *DBT*, *RA *or *RAS*. The genomic analysis demonstrated not only the activation effect of *DBT *in stimulating the proliferation and differentiation of the cultured osteoblasts but also a set of candidates of biomarkers that are specifically activated by *DBT*. These *DBT*-specific changes in gene expression may be useful in developing biomarkers for quality control of *DBT*. After identification of these DBT-specific genes and their roles, it will be easier to elucidate the action mechanism of *DBT*.

**Table 3:** Genes regulated by *DBT*, *RA *and *RAS *in cultured MG-63 cells

**Genes**	Number of genes *
Total	8064
Control	606
*DBT*-activated	883
*DBT*-specific	403
*RA*-activated	660
*RA*-specific	172
*RAS*-activated	1062
*RAS*-specific	473

*Significant changes of gene expression are defined as regulation, which can be either up-regulation when fluorescent signal in the sample was 200% greater than that of control, or down-regulation when the signal was 50% less than that of control.

## Conclusion

In verification studies of *DBT *decoction, the quality of herbal materials is ensured by authentication analysis. The ancient formula of *RA *to *RAS *ratio at 5:1 has been confirmed in both chemical composition and biological responses both *in vivo *and *in vitro*. Mechanism studies have also revealed some therapeutic effects of *DBT*. It is hoped that a systematic research and development approach, as exemplified in this article, will provide an effective method to develop Chinese herbal medicine products.

## Competing interests

The author(s) declare that they have no competing interests.

## Authors' contributions

QG and JL drafted the manuscript and did most of the experiments described in this review. JC, AC, KZ and WL assisted in the experiments. JD, AD, TD and KT helped draft and revise the manuscript. KT supervised this work. All authors read and approved the final manuscript.

## References

[B1] Tsim KWK, Kung SD, Tso, Ho (2004). Modernization of traditional Chinese medicine: problems in safety and quality assurance. Vision of 2050: Agriculture in China.

[B2] Tsim KWK (2005). *Danggui Buxue Tang *(DBT, Chinese *Angelica *Decoction): a sample trial in TCM standardization. Asia-Pacific Biotech News (APBN).

[B3] Zheng XY (2005). Pharmacopoeia of the People's Republic of China Beijing.

[B4] Sinclair S (1998). Chinese herbs: a clinical review of *Astragalus, Ligusticum *and *Schizandrae*. Altern Med Rev.

[B5] Hsiao PG (1964). Studies on the original plant and pharmacognosy of traditional Chinese medicine: Huangqi. Acta Pharm Sin.

[B6] Ma XQ, Duan JA, Zhu DY, Dong TTX, Tsim KWK (2000). Chemical comparison of *Astragali Radix *(*Huangqi*) from different regions of China. Nat Med.

[B7] Ma XQ, Duan JA, Zhu DY, Dong TTX, Tsim KWK (2000). Species identification of *Radix Astragali *(*Huangqi*) by DNA sequence of its 5S-rRNA spacer domain. Phytochemistry.

[B8] Dong TTX, Ma XQ, Clarke C, Song ZH, Ji ZN, Lo CK, Tsim KWK (2003). Phylogeny of *Astragalus *in China: Molecular evidence from the DNA sequences of 5S rRNA spacer, ITS, and 18S rRNA. J Agric Food Chem.

[B9] Zhao KJ, Dong TTX, Tu PF, Song ZH, Lo CK, Tsim KWK (2003). Molecular genetic and chemical assessment of *Radix Angelica *(*Danggui*) in China. J Agric Food Chem.

[B10] Ma XQ, Shi Q, Duan JA, Dong TTX, Tsim KWK (2002). Chemicalanalysis of Radix *Astragali *(*Huangqi*) in China: A comparison with its adulterants and seasonal variations. J Agric Food Chem.

[B11] Hu SL (1989). The Authentic and Superior Medicinal Herbals in China.

[B12] Zhu YC (1989). Plant Medicinal of Northeast China.

[B13] Zheng HZ, Dong ZH, She J (1997). Modern Study of Traditional Chinese Medicine.

[B14] Watanabe A, Araki S, Kobari S, Udo H, Tsuchida T, Uno T, Kosaka N, Shimomura K, Yamazaki M, Saito K (1998). *In vitro *propagation, restriction fragment length polymorphism, and random amplified polymorphic DNA analysis of Angelica plants. Plant Cell Rep.

[B15] Song ZY (1996). The Modern Studies on the Chinese Meteria Medica.

[B16] Wagner H, Bauer R, Xiao PG, Chen JM, Michler H (2001). Chinese Drug Monographs and Analysis: Angelica sinensis.

[B17] Lao SC, Li SP, Kan KKW, Li P, Wan JB, Wang YT, Dong TTX, Tsim KWK (2004). Identification and quantification of 13 components in *Angelica sinensis *(*Danggui*) by gas chromatography-mass spectrometry coupled with pressurized liquid extraction. Anal Chim Acta.

[B18] Song ZH, Ji ZN, Lo CK, Dong TT, Zhao KJ, Li OT, Haines CJ, Kung SD, Tsim KWK (2004). Chemical and biological assessment of a traditional Chinese herbal decoction prepared from *Radix Astragali *and *Radix Angelicae Sinensis*: orthogonal array design to optimize the extraction of chemical constituents. Planta Med.

[B19] Dong TTX, Zhao KJ, Gao QT, Ji ZN, Zhu TT, Li J, Duan R, Cheung AW, Tsim KWK (2006). Chemical and biological assessment of a Chinese herbal decoction containing *Radix Astragali *and *Radix Angelicae Sinensis*: Determination of drug ratio in having optimized properties. J Agric Food Chem.

[B20] Harlow BL, Signorello LB (2000). Factors associated with early menopause. Maturitas.

[B21] Manyonda IT, Pereira RS, Makinde V, Brincat M, Varma RT (1992). Effect of 17 beta-oestradiol on lymphocyte subpopulations, delayed cutaneous hypersensitivity responses and mixed lymphocyte reactions in post-menopausal women. Maturitas.

[B22] Bukovsky A, Presl J (1981). Control of ovarian function by the immune system: commentary on the criticisms of Schenzle. Med Hypotheses.

[B23] Gao QT, Cheung JK, Li J, Chu GK, Duan R, Cheung AW, Zhao KJ, Dong TTX, Tsim KWK (2006). A Chinese herbal decoction, *Danggui Buxue Tang*, prepared from *Radix Astragali *and *Radix Angelicae Sinensis *stimulates the immune responses. Planta Med.

[B24] Wu BC, Sun XF, Yang GY (1989). Studies on different combination of the Dang Gui Buxue decoction. Chin Med Mater Heilongjiang.

[B25] Li YK, Xu J, Zhang XC (1992). Pharmacology study on *Danggui Buxue *decoction. Chin J Herbal Pharmacol Ther.

[B26] Wu DZ, Song CQ, Hen ZF, Kong JL, Fan Y, Hu ZB (1999). Effects of *Angelicae sinensis *decoction for supplementing blood on the cardiac function in myocardial ischemia reperfusion injury of rats. Pharmacol Clin Chin Mater Med.

[B27] Ning L, Chen CX, Jin RM, Wu YP, Zhang HG, Sun CL, Song CQ, Hu ZB (2002). Effect of components of *Danggui Buxue *decoction on hematopenia. Chin J Chin Mater Med.

[B28] Mak DH, Chiu PY, Dong TTX, Tsim KWK, Ko KM (2006). *Danggui Buxue Tang *produces a more potent cardioprotective effect than its component herb extracts and enhances glutathione status in rat heart mitochondria and erythrocytes. Phytother Res.

[B29] Zhang YH, Wu GL, Jiang TL (1999). Effects of *Danggui Buxue *Decoction and the decoction-contained Serum on granulocyte-macrophage progenitor cell colony forming unit (GM -CFU). Chin J Exp Tradit Med Formulae.

[B30] Zhang YH, Wu GL, Jiang TL (1999). Effect of *Danggui Buxue *Decoction and the decoction-contained serum on erythroid progenitor cell colony forming unit (CFU-E) in mice. Chin J Exp Tradit Med Formulae.

[B31] Gao QT, Choi RC, Cheung AW, Zhu JT, Li J, Chu GK, Duan R, Cheung JK, Jiang ZY, Dong XB, Zhao KJ, Dong TT, Tsim KWK (2007). *Danggui Buxue Tang *– a Chinese herbal decoction activates the phosphorylations of extracellular signal-regulated kinase and estrogen receptor alpha in cultured MCF-7 cells. FEBS Lett.

